# Pathological Structural Alterations of Serous Cell Cilia in the Parietal Pericardium of Patients With Heart Failure Induced by Dilated Cardiomyopathy

**DOI:** 10.31083/RCM48136

**Published:** 2026-05-26

**Authors:** Yingtian Liu, Yaping Xu, Yan Chen, Yuexin Yu, Ziyu Liu, Xiangli Zhang, Bin Yang, Zhikun Guo

**Affiliations:** ^1^Henan Key Laboratory of Medical Tissue Regeneration, Xinxiang Medical University, 453003 Xinxiang, Henan, China; ^2^Henan Key Laboratory of Cardiac Reconstruction and Transplantation, Zhengzhou Seventh People's Hospital, 453000 Zhengzhou, Henan, China; ^3^Cardiac Surgery, Zhengzhou Seventh People's Hospital, 453000 Zhengzhou, Henan, China

**Keywords:** mesothelial cells, serous pericardium, histology, immunofluorescence, ultrastructure, human pericardium

## Abstract

**Background:**

This study aimed to examine pathological surface structural changes in serous cells of the pericardial parietal layer in patients with heart failure due to dilated cardiomyopathy.

**Methods:**

Pericardial tissues from five patients with dilated cardiomyopathy-induced heart failure (case group) and two heart donors (control group) were analyzed using histological methods, scanning electron microscopy (SEM), transmission electron microscopy (TEM), and immunofluorescence.

**Results:**

In both groups, mesothelial cells in the parietal pericardium were classified as flat, oval, or short columnar, typically forming a single layer, occasionally multiple layers. Most cells exhibited a brush-like border on the surface facing the pericardial cavity. A layer of flattened fibroblasts was observed beneath the basement membrane. Polygonal cells extended protrusions to contact adjacent cells. Mesothelial cells were further divided into ciliated and non-ciliated types. Most cells displayed numerous typical cilia on their surface, whereas non-ciliated cells extended processes that spanned one or more cells to connect with distant cells. TEM revealed that most ciliated mesothelial cells had uniformly arranged cilia, with visible microtubules in some. Tight junctions, intermediate junctions, and desmosomes were present along the lateral surfaces of mesothelial cells, and the basement membrane appeared uniform. Compared with normal pericardial mesothelium, mesothelial cells from patients exhibited increased numbers of cilia, ciliary edema, microtubule dissolution within cilia, and elevated expression of β-tubulin.

**Conclusions:**

Abundant cilia are present on the surface of mesothelial cells in the parietal pericardium of both healthy individuals and patients. Heart failure induced by dilated cardiomyopathy can severely damage the morphology and ultrastructure of mesothelial cilia, leading to reduced ciliary motility and impaired secretion and absorption, thereby disrupting pericardial fluid production and reflux.

## 1. Introduction

The pericardium is a membranous sac that envelops the surface of the heart. It consists of two layers: the fibrous layer and the serous layer. The fibrous layer is relatively tough, closely adheres to the parietal layer of the serous pericardium, and exhibits minimal elasticity. The serous layer is further divided into the parietal and visceral layers, with the parietal layer tightly attached to the inner surface of the fibrous layer, and the visceral layer (i.e., the epicardium) covering the heart surface. Both the parietal and visceral layers of the serous pericardium are composed of mesothelial cells, which not only secrete pericardial fluid but also play essential roles in antigen presentation, inflammation, tissue repair, coagulation, and fibrinolysis [1]. Because cilia are highly enriched in receptors, ion channels, and downstream effector molecules involved in various signaling pathways, including Hedgehog signaling and G protein-coupled receptor signaling, they play a crucial role in development and homeostasis [2]. The pericardium is crucial for maintaining cardiac function [3, 4] and is closely associated with the development and progression of several cardiac diseases [5, 6]. Given that the pericardial cavity is an enclosed space and that mesothelial cells possess both secretory and absorptive functions, the pericardium provides a unique anatomical basis for intrapericardial drug administration, which has become a focus of current research [7, 8]. Our previous studies have identified the presence of stem cells with multipotent differentiation potential in the pericardium and pericardial fluid of both rats and humans, suggesting their potential involvement in cardiac repair [9]. Although the macroscopic anatomy, morphology, and physiological functions of the pericardium have been extensively studied [10, 11], there have been no reports describing pathological surface structural changes of parietal pericardial mesothelial cells (i.e., serous cells) in patients with dilated cardiomyopathy and heart failure. Therefore, this study systematically examined the morphological characteristics of serous pericardial mesothelial cells in patients with heart failure using histological and electron microscopy techniques, focusing particularly on alterations in cilia on the cell surface. These findings aim to provide a morphological basis for further understanding the functions of the pericardium and its role in cardiac pathology.

## 2. Materials and Methods

### 2.1 Basic Information of Patients and Donors and Pericardial Tissue Samples

Parietal pericardial tissues were obtained from five patients with dilated cardiomyopathy and two heart transplant donors. The patients’ pericardial tissues constituted the case group, while the donors’ pericardial tissues served as the normal control group. Two patient samples and one normal sample were used for transmission electron microscopy (TEM), while the remaining five patient samples and one normal sample were used for histological analysis and scanning electron microscopy (SEM). All human pericardial samples were collected from patients who underwent heart transplantation at the Affiliated Zhengzhou Seventh People’s Hospital of Xinxiang Medical University. Written informed consent was obtained from all participants, and the study was approved by the Ethics Committee of Zhengzhou Seventh People’s Hospital in accordance with the Declaration of Helsinki (Approval No.: 2024[ky-008]). Tissue blocks were excised from the edges of the pericardial incision.

The patient cohort consisted of three males and two females aged 49–64 years (mean age: 56.8 years). The two donors were healthy males aged 32 and 38 years, respectively. The disease duration ranged from 10 to 16 years, with a mean of 12.8 years. Heart failure in all patients was induced by primary (hereditary) dilated cardiomyopathy. The ultrasound diagnosis met the criteria for heart failure. According to the New York Heart Association (NYHA) classification of cardiac function, four cases were classified as class IV and one case as class III (see Table 1).

**Table 1.** **Basic information of heart transplant recipients and donors**.

No	Age	Gender	Group	DC (year)	LVEF (%)	LVEDV (mL)	Classes
01	55	M	HF	16	28	401	IV
02	64	F	HF	10	20	168	IV
03	59	M	HF	10	28	305	IV
04	49	F	HF	13	25	303	III
05	57	M	HF	15	17	356	IV

Note: HF, heart failure; DC, duration of disease; LVEF, left ventricular ejection fraction; LVEDV, left ventricular end-diastolic volume.

### 2.2 Histological Sectioning and Staining

Fresh pericardial tissue samples (1 cm × 2 cm) from five patients and one normal control were collected and fixed in 4% paraformaldehyde for 48 hours. The samples were then processed using standard paraffin embedding techniques and sectioned at a thickness of 5 µm. Sections were stained with hematoxylin and eosin (H&E) and mounted with neutral resin. The prepared slides were examined and analyzed under an optical microscope for morphological evaluation.

### 2.3 Transmission Electron Microscopy

Fresh pericardial tissues from two patients and one normal control were cut into 1 mm × 1 mm sections and immediately fixed in 4% glutaraldehyde prepared in phosphate-buffered saline (PBS). The samples were refrigerated at 4 °C for 48 hours. Following primary fixation, the tissue sections were subjected to standard procedures, including secondary fixation, dehydration, and embedding, followed by semi-thin sectioning. Mesothelial cells were localized using Azan staining. Ultra-thin sections were then prepared and stained at the selected sites. Observations and analyses were performed using a Hitachi HT7800 transmission electron microscope (HT7800; Hitachi High-Tech Corporation, Tokyo, Japan).

### 2.4 Scanning Electron Microscopy

Human pericardial tissues measuring 2 cm × 2 cm were fixed in 2.5% glutaraldehyde for 24 hours. The samples were then rinsed with 0.1 mol/L PBS (pH 7.2) for 1 hour, followed by dehydration through a graded ethanol series. After dehydration, the tissues were dried using a critical point dryer. The dried samples were mounted on stubs with conductive adhesive tape and sputter-coated with gold for 1 minute using an ion sputter coater. The specimens were then observed and photographed using a Zeiss EVO-10 scanning electron microscope (EVO-10; Carl Zeiss AG, Oberkochen, Baden-Württemberg, Germany).

### 2.5 Immunofluorescence and Wheat Germ Agglutinin (WGA) Staining

Fresh pericardial tissues measuring 1 cm × 2 cm were cryo-embedded and sectioned at a thickness of 8 µm. The sections were fixed in cold acetone at 4 °C for 10 minutes, followed by washing with PBS. After blocking with 10% goat serum at 37 °C for 30 minutes, sections were incubated overnight at 4 °C with the primary antibody (rabbit anti-β-tubulin antibody, Affinity, 1:500). Following PBS washing, sections were incubated at room temperature for 1 hour with the fluorescent secondary antibody (Cy3-conjugated goat anti-rabbit IgG, Beyotime, 1:500), and washed again with PBS. Subsequently, IF488-WGA working solution (Ruibao Biotechnology, 1:250) was applied, followed by DAPI staining for 10 minutes. After final PBS washing, sections were photographed under a fluorescence microscope. Ten visual fields containing mesothelial cells were randomly selected from each section, and the positive area (expressed as a percentage of the total area) was calculated for each field. Data are expressed as mean ± standard deviation (SD), and comparisons between groups were performed using a *t*-test. A value of *p* < 0.05 was considered statistically significant.

### 2.6 Detection of Inflammatory Factors in Pericardial Fluid

After extraction of the donor and recipient hearts, 10 mL of pericardial fluid was immediately collected, and 10 inflammatory factors were measured using flow cytometry, including interleukin-10 (IL-10), interleukin-12p70 (IL-12p70), interleukin-17 (IL-17), interleukin- 1 beta (IL-1β), interleukin-6 (IL-6), interleukin-8 (IL-8), interferon-alpha (IFN-α), interferon-gamma (IFN-γ), tumor necrosis factor-alpha (TNF-α), and interleukin-5 (IL-5). The results were analyzed using Prism 7.00 statistical software (7.00; GraphPad Software, Inc., San Diego, CA, USA). A *t*-test was used to compare data between the two groups, and the results are expressed as the mean ± SD ($\bar{X}$ ± SD). A *p* value < 0.05 was considered statistically significant.

## 3. Results

### 3.1 Histological Structure of the Parietal Layer of the Serous Pericardium

**Fig. 1.**
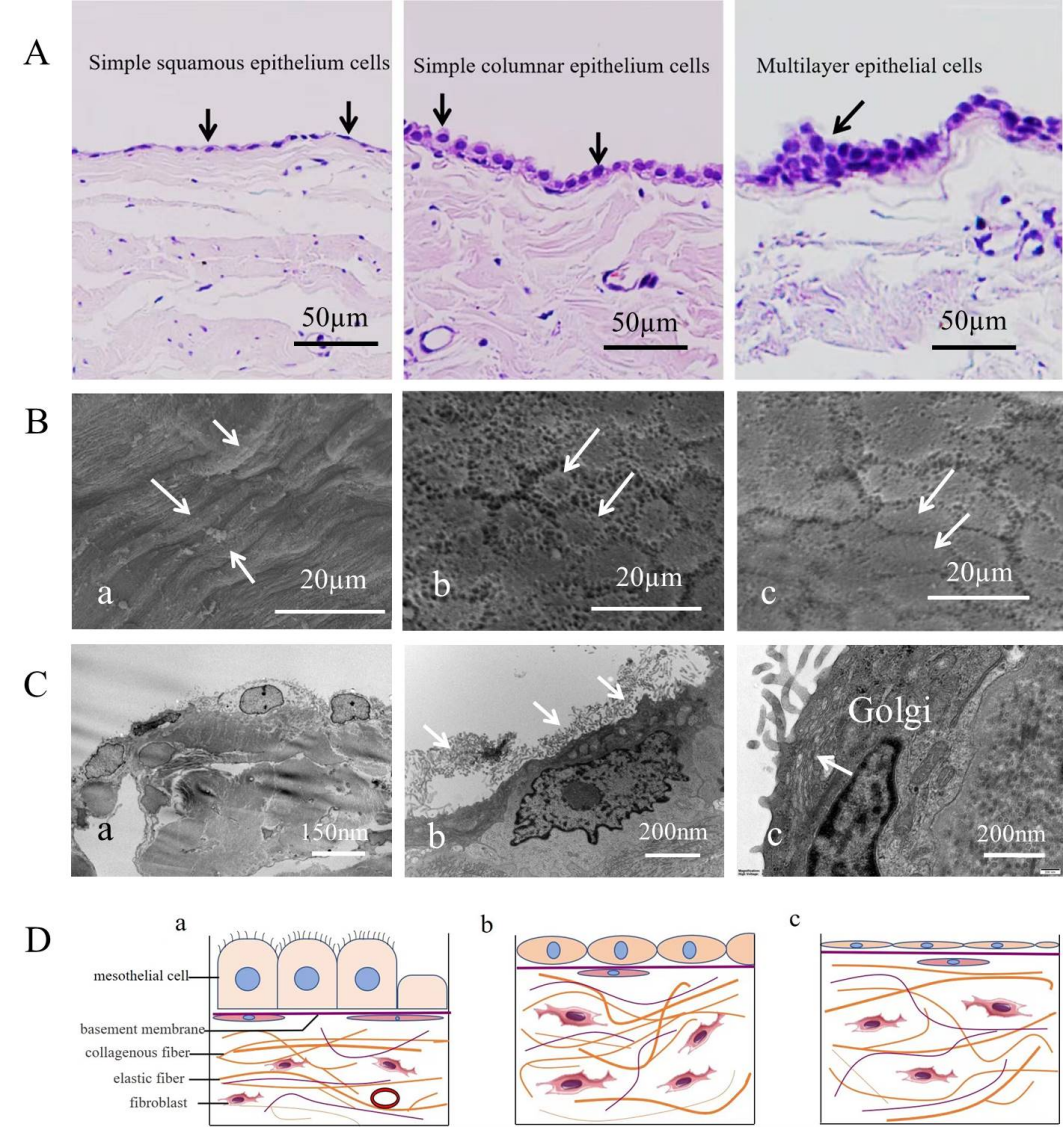
**Classification and arrangement of human parietal serous cells**. (A) Three types of arrangements: a single layer of flat cells (i.e., squamous epithelial cells), a single layer of columnar cells, and a multilayered arrangement of cells (indicated by arrows), scale bar = 50 µm. (B) Scanning electron microscopy image of the serous cell surface. Arrows in (a) indicate long spindle-shaped cells; arrows in (b) indicate polygonal cells; arrows in (c) indicate oval cells. scale bar = 20 µm. (C) Transmission electron microscopy image of serous cells. (a) Flat and oval mesothelial cells in normal tissue, scale bar = 150 nm. (b) Numerous microvilli are observed on the surface of patient serous cells (arrows), scale bar = 200 nm. (c) Serous cells contain numerous Golgi apparatus (arrows), scale bar = 200 nm. (D) Schematic representation of different mesothelial cell morphologies and their spatial relationships. (a) Columnar mesothelial cells rich in cilia on the cell surface. (b) Elliptical mesothelial cells without cilia on the cell surface. (c) Flat cells without cilia. Three different forms of cells have a complete basement membrane at the base, with a layer of flat fiber cells dispersed below the basement membrane.

**Fig. 2.**
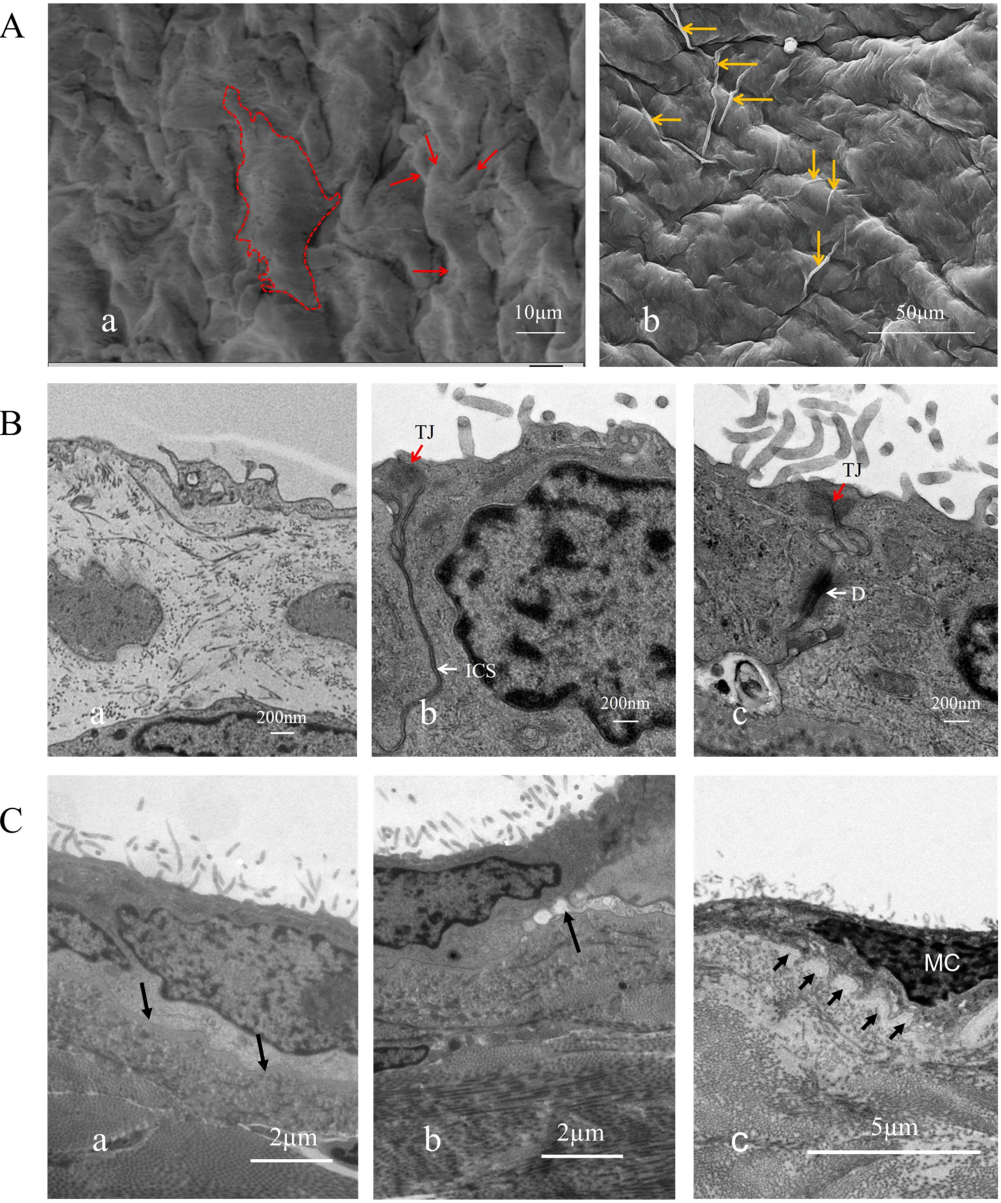
**Surface ultrastructural morphology and structure of human parietal serous cells**. (A) Serous cells at the cardiac base were observed under scanning electron microscopy. These cells lack cilia but display abundant surface protrusions. (a) Within the dashed outline is a complete mesothelium with multiple protrusions. The arrow indicates the cellular protrusion, scale bar = 10 µm. (b) A displays long protrusions spanning one or several cells (indicated by arrows), scale bar = 50 µm. (B) Connections between serous cells and pathological alterations of cilia, scale bar = 200 nm. (a,b) show normal serous cells with fewer cilia, while (c) shows patient-derived cells with increased cilia. (C) Basement membrane (arrow). (a) shows an intact, smooth basement membrane in the normal sample, scale bar = 2 µm; (b) shows a disrupted basement membrane in the patient sample, scale bar = 2 µm; (c) shows mesothelial cells from the patient penetrating into the basement membrane, leading to discontinuity, scale bar = 5 µm. a, b, and c all show a large number of disordered cilia on the cell surface. D, Desmosomes (white arrows); TJ, tight junctions (red arrows); ICS, intermediate junctions (white arrows).

**Fig. 3.**
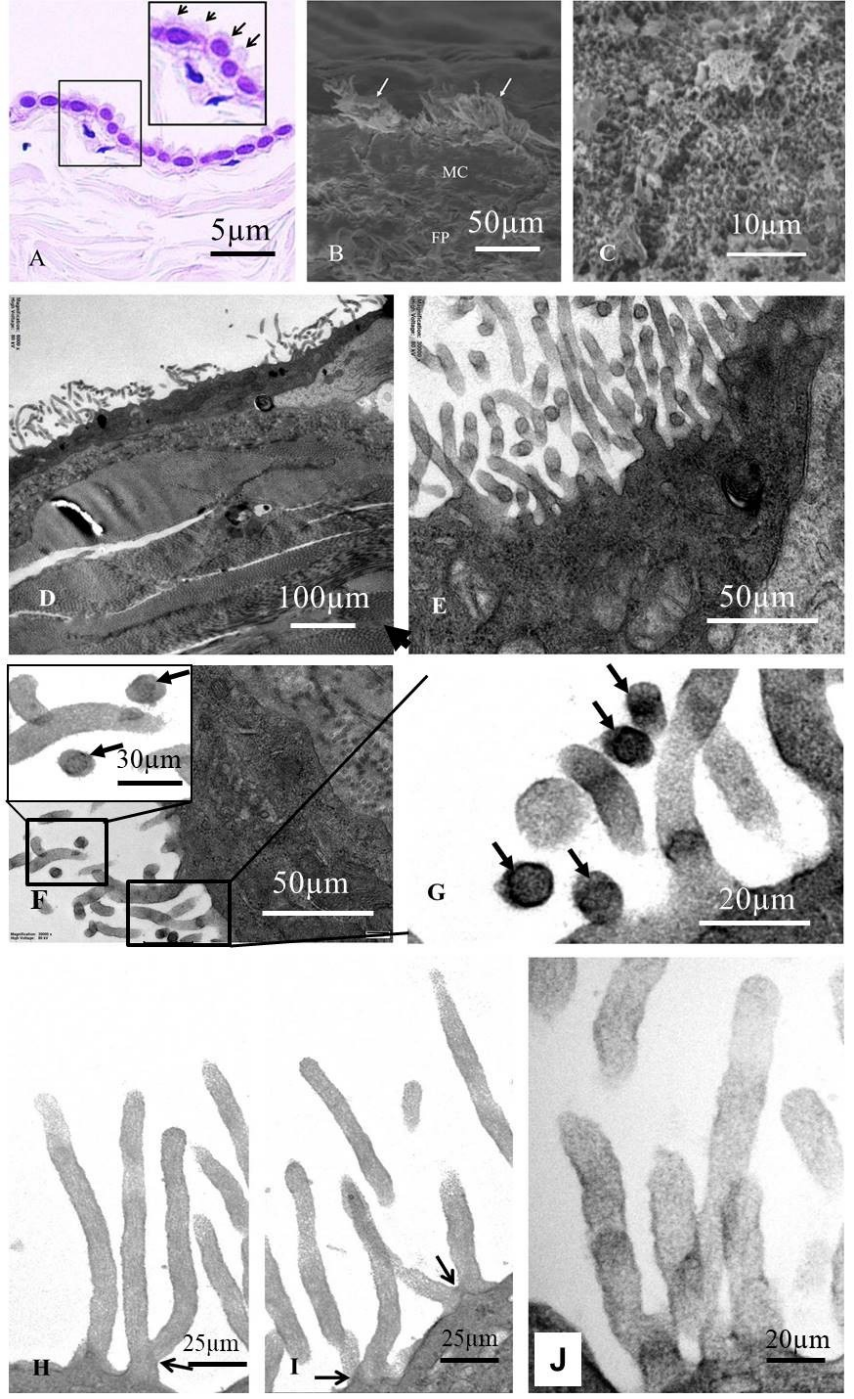
**Pathological changes of cilia in serosal cells**. (A) The surface of serosal cells shows a brush border under light microscopy (hematoxylin and eosin [H&E] staining, indicated by arrows), scale bar = 5 µm. (B) Clustered cilia on the cell surface were observed by scanning electron microscopy (arrows), scale bar = 50 µm. (C) Ciliary processes of varying lengths were observed by scanning electron microscopy, scale bar = 10 µm. (D) Normal cell cilia, scale bar = 100 µm. (E) Cilia of patient-derived cells, arrow indicates the cross-section of cilia, scale bar = 50 µm. (F,G) Microtubule-containing cilia (arrows) and microtubule-deficient cilia, which are generally thicker and have a blurred internal matrix, F, scale bar = 30 µm (high mag), scale bar = 50 µm (low mag), G, scale bar = 20 µm. (H,I) Bifurcated cilia (arrows), scale bar = 25 µm. (J) Cilia exhibiting increased stromal density and edema, scale bar = 20 µm. (D–J) Observed under transmission electron microscopy.

Under both light and electron microscopy, there were no significant differences in the tissue or cellular morphology of the parietal pericardium between patients and normal controls. The main structural features were as follows: The mesothelial cells of the serous pericardium were classified as flat, oval, or short columnar in shape. These cells were typically arranged in a single layer, although in some regions, two to four overlapping cells were observed, forming a multilayered arrangement. Most cells exhibited a brush border on the surface facing the pericardial cavity. The nuclei were centrally located and their shapes corresponded to the overall morphology of the cells, appearing flat, round, or oval. The region containing the nucleus was often slightly elevated compared with the surrounding cell surface. The basal surface of the mesothelial cells was supported by a thin basement membrane, beneath which an occasional layer of flattened fibroblasts was present. Below the basement membrane lay the fibrous pericardium, characterized by abundant collagen fiber bundles and a sparse distribution of fibroblasts (Fig. 1A,C; Fig. 2C,a; Fig. 3A).

### 3.2 Surface Morphological Characteristics and Interrelationship of Parietal Serous Cells

Scanning electron microscopy revealed that the serous cells of the parietal layer of the pericardium in both the case group and the normal group exhibited three distinct surface morphologies: spindle-shaped, elliptical, and polygonal (Fig. 1B). Spindle-shaped cells were arranged in parallel, whereas oval-shaped cells were often scattered among other cell types. Polygonal cells, mostly hexagonal, extended small projections that made contact with neighboring cells. The surfaces of polygonal cells displayed numerous finger-like protrusions, while the surfaces of spindle-shaped and oval cells were smooth and devoid of such structures (Fig. 1A,D; Fig. 2A,a,b). Cells with smooth surfaces exhibited central prominences and peripheral extensions of larger cellular projections, resulting in diverse morphologies that often resembled starfish or twisted shapes. These larger protrusions facilitated connections between adjacent cells, with some extensions spanning over one or more cells to establish contact with distant cells (Fig. 2A). Finger-like cilia were present on the surfaces of all three cell types, although their numbers varied (Fig. 2B). Therefore, pericardial serosal cells can be categorized into ciliated and non-ciliated cells. Moreover, the cilia exhibited uneven lengths and irregular arrangements (Fig. 2C; Fig. 3E–J).

Mesothelial cells displayed tight junctions, intermediate junctions, and desmosomal structures along their lateral surfaces (Fig. 2B). In regions lacking specialized junctional complexes, protrusions were observed (Fig. 3C). A uniformly textured basement membrane was present beneath the basal side of the mesothelial cells, under which smaller fibroblasts were identified—consistent with observations under light microscopy (Fig. 1C,a,b). The contact between the basement membrane and mesothelial cells was either smooth or interdigitated in a staggered pattern (Fig. 2C,c). Within the mesothelial cells, an abundant Golgi apparatus and vesicular structures were observed, whereas mitochondria appeared relatively underdeveloped (Fig. 1C,c). These findings provide detailed insights into the ultrastructural features of mesothelial cells in the serous pericardium, elucidating their morphology, junctional complexes, and organelle characteristics.

Overall, no significant differences were observed in the morphological characteristics of serous cells between normal individuals and heart failure patients; the primary pathological changes were confined to the cilia.

### 3.3 Pathological Changes of Cilia in Serosal Cells

Under the light microscope, a brush-like border was observed on the surface of both normal and patient serosal cells (Fig. 3A). Scanning electron microscopy revealed clustered cilia on the cell surface, which varied in length (Fig. 3B,C). Transmission electron microscopy showed that mesothelial cells were mainly flat or oval in shape. Since the cells were examined in cross-section, short cylindrical cells were not observed. Most mesothelial cells displayed uniformly fine protrusions on their surfaces (Fig. 1C,D; Fig. 2B,C; Fig. 3D). In cross-sections of these protrusions, microtubules were arranged circumferentially beneath the cell membrane, with two or three parallel microtubules located at the center of the protrusion (arrows in Fig. 3F–H). However, only a few sections showed typical microtubule structures; most lacked visible microtubules. In longitudinal sections of the protrusions, no obvious microtubules were detected. In both transverse and longitudinal sections, the internal matrix of cilia without microtubules appeared blurred (Fig. 3E–J). Overall, the patient’s cell processes contained few or absent microtubules. Some cilia branched at their bases (arrows in Fig. 3H,I), whereas other cells exhibited smooth surfaces with few or no cilia. Notably, only a small portion of the cilia on the patient’s cell surface showed clearly defined microstructures, while most lacked visible internal organization. Cilia with unclear internal structures appeared thicker, suggesting interstitial expansion, tubulin dissolution, and edema (Fig. 3E–J).

Compared with the normal group, mesothelial cells from patients exhibited an increased number of cilia, which were longer and more sparsely distributed (Fig. 1C, a,b). Electron microscopy revealed that many cilia became thicker, with blurred microtubules and signs of edema. In some regions, numerous serosal cells lacked cilia altogether. Cells without cilia displayed more rough surface protrusions, particularly longer intercellular extensions that enhanced connections among adjacent or neighboring cells.

### 3.4 Pathological Expression of β-Tubulin in Parietal Serous Cells

**Fig. 4.**
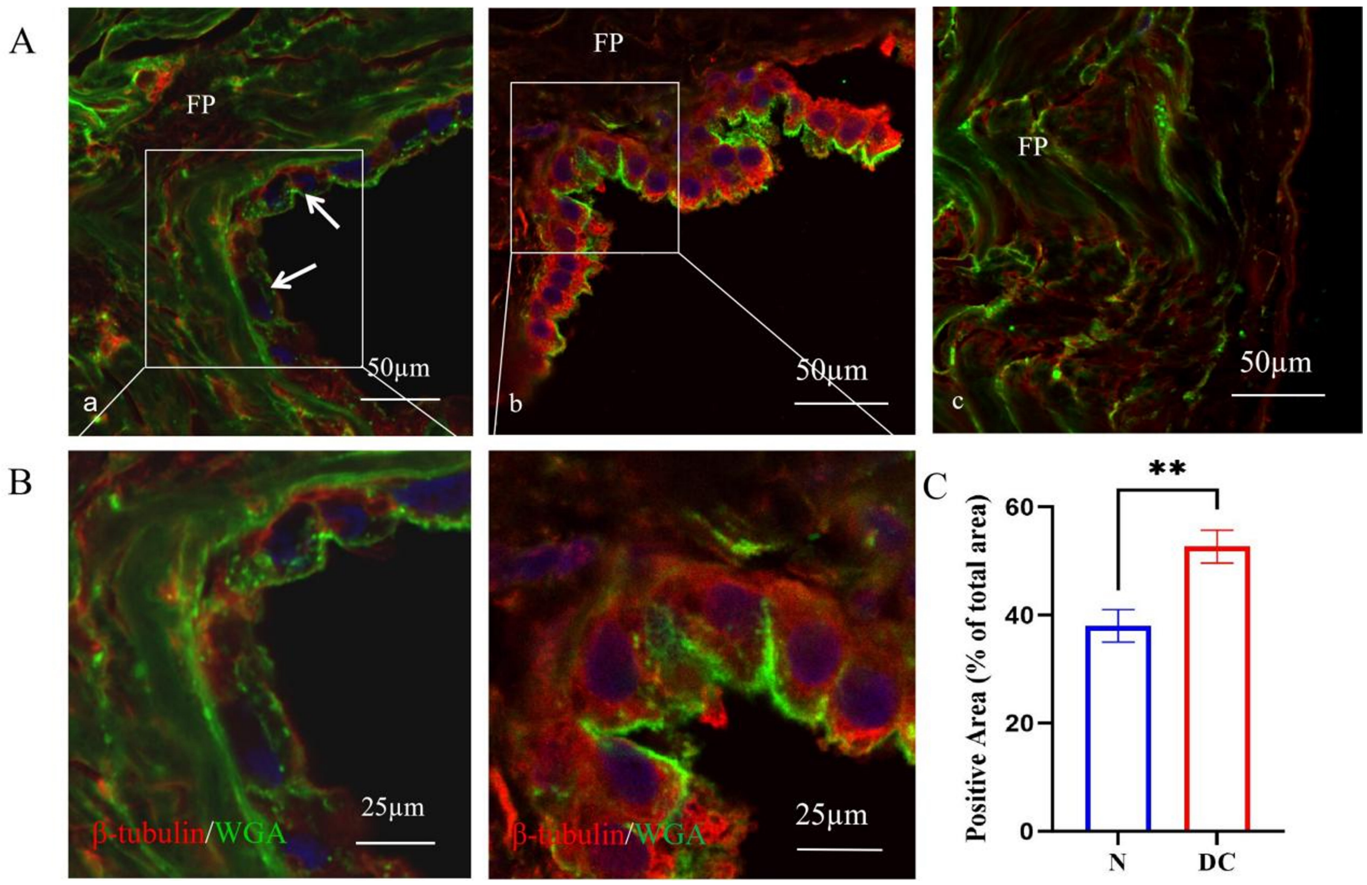
**Differential expression of β-tubulin between normal and patient serous cells (arrows)**. (A) Red fluorescence indicates β-tubulin, stained by immunofluorescence; green fluorescence marks the cell membrane, stained with WGA, scale bar = 50 µm. (a) Normal parietal serous cells. Arrows indicate serous cell membrane; (b) Serous cells from patients with dilated cardiomyopathy; (c) Fibrous pericardium (EP). (B) High-magnification views of images (A,a) and (A,b), scale bar = 25 µm. (C) Quantitative comparison of β-tubulin expression in serous cells between normal and dilated cardiomyopathy (DC) groups. N, normal. **p < 0.01.

Immunofluorescence staining revealed strong positive expression of β-tubulin in the parietal serous cells of patients, whereas normal parietal serous cells showed weak positive or even negative expression. Under high magnification, β-tubulin was evenly distributed throughout the cytoplasm, with occasional visible cross-sections of microtubules. A significant difference in expression intensity was observed between the two groups (*p* < 0.01). WGA staining was applied to visualize the cell membrane, allowing clear localization of β-tubulin within the cytoplasm. It is noteworthy that a small amount of β-tubulin was also detected in various cells of the fibrous pericardium (Fig. 4).

### 3.5 Detection Results of Inflammatory Factors in Pericardial Fluid

Compared with normal pericardial fluid, the levels of IL-10, IL-6, IL-8, and TNF-α were significantly increased (*p* < 0.05 or *p* < 0.01), whereas the levels of IL-12p70, IL-17, IL-1β, IL-5, IFN-α, and IFN-γ showed no significant differences (*p* > 0.05). The results are presented in Table 2.

**Table 2.** **Comparison of inflammatory factors in pericardial fluid between patients with heart failure and normal individuals (unit: pg/mL; mean ± SD)**.

	IL-10	IL-12P70	IL-17	IL-1β	IL-5	IL-6	IL-8	IFN-α	IFN-γ	TNF-α
HF	29.2 ± 15.7	2.5 ± 1.4	12.3 ± 7.4	5.5 ± 4.0	1.0 ± 0.3	1119.9 ± 321.2	3587.4 ± 2110.0	1.3 ± 0.6	2.3 ± 0.6	90.0 ± 119.4
Nm	20.1 ± 1.03	2.4 ± 0.8	12.0 ± 2.3	5.0 ± 1.9	1.1 ± 0.2	98.21 ± 20.1	102.32 ± 13.2	1.2 ± 04	2.4 ± 0.4	48.32 ± 6.4
*p*	<0.05	>0.05	>0.05	>0.05	>0.05	<0.05	<0.01	>0.05	>0.05	<0.01

Note: HF, heart failure; Nm, normal pericardial fluid; IL-10, interleukin-10; IL-12p70, interleukin-12p70; IL-17, interleukin-17; IL-1β, interleukin- 1 beta; IL-5, interleukin-5; IL-6, interleukin-6; IL-8, interleukin-8; IFN-α, interferon-alpha; IFN-γ, interferon-gamma; TNF-α, tumor necrosis factor-alpha. Compared with normal pericardial fluid, *p* < 0.05 shows a significant difference.

## 4. Discussion

The serous pericardium consists of visceral and parietal layers, both composed of a single layer of epithelial cells known as mesothelial cells. In this study, we examined the parietal serosa of healthy individuals and patients with heart failure secondary to dilated cardiomyopathy, focusing on the pathological changes in parietal serous cells under heart failure conditions. Traditionally, these cells have been described as squamous, with round or oval nuclei that are centrally located and protrude toward the cell surface. However, detailed descriptions of their surface morphology are limited in the literature [12, 13, 14]. This study revealed morphological diversity among mesothelial cells in the parietal layer of the human serous pericardium. Based on surface features, cells can be divided into ciliated and non-ciliated types. According to their morphology, they can be classified as flat, columnar, or oval, with columnar cells being less common. These morphological variations may reflect different functional states. Although most mesothelial cells form a single layer, some regions exhibited multilayered arrangements, reaching up to four layers in thickness. The functional differences between monolayered and multilayered mesothelial cells warrant further investigation.

The boundaries between mesothelial cells are convoluted, and adjacent cells frequently overlap. These cells possess well-developed junctional complexes, including tight junctions, adherens junctions, gap junctions, and desmosomes, consistent with previous reports and our observations. In addition, an irregularly distributed basement membrane was observed at the basal surface of mesothelial cells, beneath which small, flat, fibroblast-like cells were sometimes present. The morphological reason for the regular arrangement of these fibroblast-like cells remains unclear. These features were observed in both normal individuals and patients with dilated cardiomyopathy, suggesting that the principal pathological alterations in heart failure are localized to the cilia rather than to overall cell morphology.

Under light microscopy, a brush-like border was observed on the surface of some serous cells. The fine surface projections of pericardial mesothelial cells, previously described qualitatively as microvilli, were found in this study—by transmission electron microscopy—not to be microvilli [13, 14, 15]. Instead, they exhibited the ultrastructural characteristics of cilia: centrally located with two parallel microtubules surrounded by nine peripheral pairs arranged circularly beneath the cell membrane. Cilia are uncommon cellular structures during cardiac embryonic development. They are known to facilitate motion or fluid transport and are typically found in the respiratory tract, reproductive tract, brain ventricles, and spinal canal (e.g., ependymal cells) [16, 17, 18, 19, 20]. In adults, only a few peritoneal mesothelial cells possess cilia [12]. Ishihara *et al*. [21] provided detailed descriptions of cilia on parietal serous cells in healthy individuals, confirming their ciliary rather than microvillar nature. The present study supports this finding and further demonstrates that, in patients with heart failure, pathological changes are concentrated in the cilia. Specifically, cilia exhibited an increased number, swelling, and dissolution of their microtubular system, resulting in a blurred internal microstructure. Immunopathological analysis confirmed that β-tubulin expression in mesothelial cells was elevated compared with that in normal cells. This may result from the accumulation or precipitation of microtubule proteins following microtubule dissolution within the cilia. Such pathological alterations likely lead to reduced ciliary motility. The observed increase in cilia number may represent a compensatory response to microtubule degeneration. To further explore the pathological mechanisms underlying ciliary damage, ten inflammatory factors were assessed in this study. Among them, IL-10, IL-6, IL-8, and TNF-α were significantly elevated compared with the normal group. These findings suggest that ciliary edema and dissolution are likely closely associated with inflammatory factors.

Cilia are broadly classified as motile or non-motile (primary or sensory) [22]. Primary cilia are present throughout cardiac development [17, 18] and play crucial roles in cellular signaling, embryonic morphogenesis, organogenesis, and the maintenance of tissue homeostasis [2, 19, 20]. They are found in rat embryos, neonates, and juvenile hearts but disappear in mature myocardium [23], suggesting that ciliary function is largely confined to early cardiac development and only limited in adult tissue. The samples in this study were derived from adult hearts, indicating that cilia on the surface of adult pericardial mesothelial cells represent remnants of embryonic cilia. Recent studies suggest that cilia may reappear and participate in pathological processes such as cardiac fibrosis and regeneration [24]. Previous investigations on cardiac cilia have focused mainly on lower vertebrate models or human cell lines during development, whereas data on ciliary alterations in adults remain scarce. To our knowledge, this study provides the first evidence of ciliary changes in the human pericardium associated with heart failure.

For many years, mesothelial cells were thought to be morphologically uniform and described simply as flattened, scale-like cells [12] or hexagonal or elongated cells [24]. However, such generalizations fail to account for regional or functional variations. Our scanning electron microscopy analysis demonstrated considerable diversity among mesothelial cells in the parietal serous pericardium. These cells exhibited hexagonal and flat, star-shaped (elliptical), or twisted (elongated) forms. Based on the presence or absence of cilia, they can be categorized into ciliated and non-ciliated cells. Ciliated cells predominantly appeared on flat surfaces, whereas non-ciliated cells displayed smooth surfaces with two types of projections: (1) larger projections that directly connect to neighboring cells, and (2) slender extensions that span one or more cells to establish contact with distant cells. This complex configuration may enhance both mechanical linkage and intercellular communication. Although this interpretation remains speculative, it highlights a potential new direction for future research.

Although the internal ultrastructure of pericardial mesothelial cells was not the primary focus of this study, several features consistent with previous reports were observed. A notable characteristic was the presence of numerous vesicles and vacuoles, reflecting the cells’ biosynthetic activity and their potential role in fluid and particle transport across the serous membrane [13, 15]. Mesothelial cells also contained abundant Golgi apparatus and rough endoplasmic reticulum, although their mitochondria were fewer in number compared to cardiomyocytes.

In summary, this study systematically examined the morphological and pathological characteristics of parietal serous cells in normal individuals and in patients with heart failure secondary to dilated cardiomyopathy. The results demonstrated that human parietal serous cells exhibit diverse morphologies and can be categorized into ciliated and non-ciliated types. In heart failure, the major pathological alterations include increased cilia number, ciliary edema, microtubule dissolution within cilia, and elevated microtubule protein expression. These changes collectively lead to impaired ciliary motility, secretion, or absorption function.

### Limitations

The limitation of this study lies in the small sample size used, as obtaining fresh human hearts is difficult, and there is no comparison with heart failure induced by hypertrophic cardiomyopathy and restrictive cardiomyopathy. In addition, if a large animal model of heart failure is prepared and some intervention factors are applied before observing the morphological changes of cilia in pericardial mesothelial cells, it will provide more powerful evidence for further elucidating the mechanism of cilia pathological changes.

## 5. Conclusions

The surface of mesothelial cells in the serosal parietal pericardium of humans is rich in cilia, greatly increasing the surface area of the cells. Dilated cardiomyopathy induced heart failure can severely damage the morphology of mesothelial cilia, leading to cilia edema, dissolution or disappearance of microtubule proteins inside, resulting in reduced ciliary movement, impaired secretion and absorption, thereby disrupting the production and reflux of pericardial fluid.

## Availability of Data and Materials

The datasets used and analyzed during the current study are available from the corresponding author on reasonable request.
